# Disseminated Candidiasis in a Young, Previously Healthy, Dog and Review of Literature

**DOI:** 10.1007/s11046-016-0092-6

**Published:** 2016-11-30

**Authors:** Nicole Willems, Dirk J. Houwers, Yvette M. Schlotter, Bart Theelen, Teun Boekhout

**Affiliations:** 1 Department of Clinical Sciences of Companion Animals, Faculty of Veterinary Medicine, Utrecht University, Yalelaan 108, 3584 CM Utrecht, The Netherlands; 2 Department of Infectious Diseases and Immunology, Faculty of Veterinary Medicine, Utrecht University, Yalelaan 1, 3584 CL Utrecht, The Netherlands; 3 Department of Yeast and Basidiomycete Research, CBS Fungal Biodiversity Centre, Uppsalalaan 8, 3584 CT Utrecht, The Netherlands

**Keywords:** Disseminated candidiasis, Candida, Albicans, Dog, Mycosis, Yeasts, Hyphae

## Abstract

**Background:**

The reports on disseminated candidiasis in dogs so far describe at least one predisposing factor. This case report, however, highlights candidiasis in a dog without any known predisposition.

**Patient:**

A 1.5-year-old intact female Hovawart dog was presented with subcutaneous nodules and polyuria/polydipsia. An excisional biopsy revealed a chronic pyogranulomatous and necrotizing inflammation with mycotic structures. The patient became febrile and lethargic, and developed lameness.

**Methods:**

A physical examination, blood tests, urinalysis, thoracic radiographs, abdominal ultrasonography of the abdomen, fine-needle aspiration biopsies, and a culture of a subcutaneous nodule aspirate were obtained. Selected sections of multiple organs were collected for routine histology postmortem. The isolate and a subcutaneous mass were subjected to molecular identification and matrix-assisted laser desorption/ionization time-of-flight mass spectrometry (MALDI-TOF–MS) analysis.

**Results:**

Clinical, laboratory, and radiological findings were consistent with a granulomatous chronic systemic inflammation. Cytology and histology showed a pyogranulomatous and necrotizing inflammation with myriads of intra- and extra-cellular yeasts and extracellular hyphae. Culture yielded numerous yeast colonies, which appeared *Candida albicans*–like, but showed a negative serum test and a low identification in API 20 C AUX. Nucleic acid sequences showed homology with the *C. albicans*-type strain CBS 562. Multilocus sequence typing (MLST) resulted in a new type with designation DST121. The identification of the isolates was confirmed by MALDI-TOF–MS analysis.

**Conclusion and Clinical Importance:**

Future MLST typing and investigation of virulence can provide further evidence whether this MLST-type is associated with clinical cases of disseminated candidiasis without an apparent predisposing condition.

A 1.5-year-old intact female Hovawart dog was initially presented to a veterinary practitioner with subcutaneous nodules and polyuria/polydipsia. An excisional biopsy revealed a chronic pyogranulomatous and necrotizing inflammation with mycotic structures. The patient became febrile and lethargic, developed lameness of the right hind limb, and therefore was referred to the Department of Clinical Sciences of Companion Animals of the Faculty of Veterinary Medicine in Utrecht. On admission, the dog had multiple subcutaneous nodules, varying in size from 2 mm to 2 cm, mostly in the abdominal region, on the thoracic wall, and the medial site of the right stifle joint, together with swelling of the right metatarsal region and enlargement of the popliteal lymph node. A 4 × 4 × 4 cm mass was palpable on the caudomedial side of the left mandibular branch. A second mass, approximately 8 × 10 × 8 cm, was palpable in the dorsal part of the mesogastrium. Complete blood count revealed a leukocytosis, with an increase in segmented neutrophils and monocytes and a slightly decreased hematocrit value. Serum biochemical analysis showed an increase in alkaline phosphatase, total protein, and beta globulin levels and a balancing decrease in the albumin level. These results were consistent with a granulomatous chronic systemic inflammation. Abdominal ultrasonography revealed enlargement of both kidneys and an irregular hypoechoic structure in the left renal cortex. These findings, in combination with proteinuria, hemoglobinuria, and hematuria, were indicative for nephropathy. In addition, the large palpable mass in the mesogastrium presented as a large hypoechoic lobular mass surrounding the caudal vena cava. The spleen was enlarged and thrombosis was suspected in multiple splenic veins. A second hypoechoic mass 1.8 × 3.3 × 1.5 cm in the region of the mesenteric lymph nodes was also present. Finally, the distal part of the 3rd metatarsal bone of the right hind limb showed irregular bone margins and soft tissue swelling. Thoracic radiographs revealed sternal lymphadenopathy. Fine-needle aspiration biopsies were taken from the enlarged popliteal lymph node, a subcutaneous nodule on the thoracic wall, and the abdominal mass in the epigastrium. Cytology showed a pyogranulomatous and necrotizing inflammation with fungal hyphae and yeasts. Culture of a subcutaneous nodule aspirate yielded numerous yeast colonies, which appeared *Candida albicans*—like, but showed a negative serum test and a low identification in API 20 C AUX. A tentative diagnosis of systemic candidiasis was made and because of the advanced stage and poor prognosis, the owner elected euthanasia. A complete necropsy was performed. The subcutis contained dozens of masses varying from several millimeters to centimeters in size, some extending into the underlying muscular layers, with a whitish smooth cut surface. The architecture of the mass caudomedial of the left mandibular branch and the irregular, multinodular mass between the stomach and spleen was disturbed to such an extent that preexisting tissue was no longer recognizable. Based on their anatomical position, the masses most likely originated from lymph nodes. The skin, kidneys (Fig. 1a, b), stomach, heart, peritoneum, and mesentery showed severe disruption of normal architecture by multifocal to coalescing similar masses. The lesion in the right hind limb affected the distal portion of the 3rd metatarsal bone. Selected sections of multiple organs were subjected to routine histology. The masses consisted of granulomas with a central core of necrosis surrounded by peripheral neutrophils, followed by epithelioid macrophages and multinucleated giant cells, bordered by a layer of fibroblasts intermixed with moderate numbers of lymphocytes and plasma cells. Within the granulomas, myriads of intra- and extra-cellular yeasts and extracellular hyphae were detected (Fig. 1c).

**Fig. 1:**
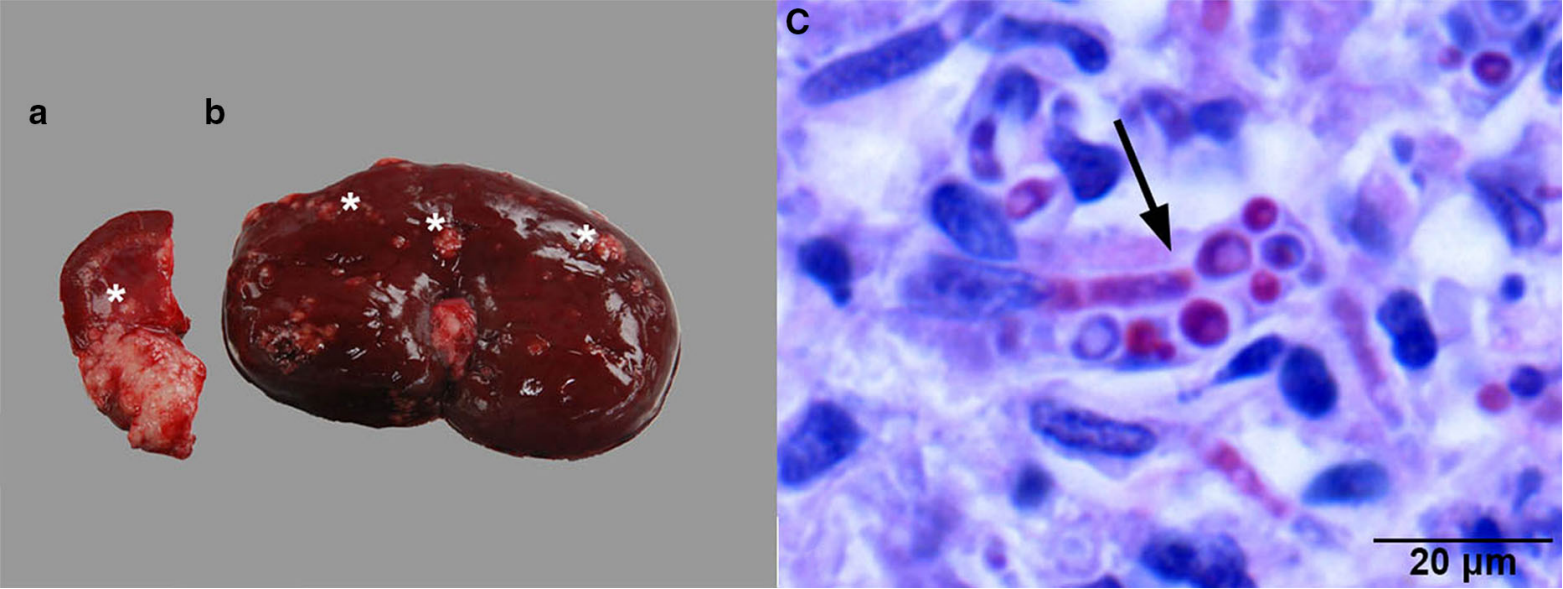
**a**, **b** A macroscopic sagittal section of the left kidney showing disruption of normal architecture of the medulla (**a**) and cortex (**b**) by multifocal to coalescing masses (*asterisks*). **c.** A representative microscopic image of a granuloma in the left kidney with fungal elements (*arrow*). *Hematoxylin and eosin (H&E) stain*

The isolate and one of the subcutaneous masses in paraffin were subsequently subjected to molecular identification. For the isolate, this was achieved by extracting DNA using the UltraClean™ Microbial DNA Isolation Kit (Mo Bio Laboratories inc., Carlsbad, USA) according to the instructions of the manufacturer. Fragments containing the ITS1 + 5.8S + ITS2 region were amplified using primers LS266 and V9G [1]. Fragments containing the LSU region were amplified using primers LR0R and LR5 [2]. For the paraffin-embedded subcutaneous masses, DNA was obtained using the QuickExtract™ FFPE DNA Extraction Kit (Epicenter, Madison, USA). The ITS1 and ITS2 parts of the internal transcribed spacer (ITS) region of the ribosomal DNA were amplified and sequenced using primers ITS1 and ITS2, and ITS3 and ITS4, respectively. The nucleic acid sequence of both strands of both PCR products was determined using ABI Prism^®^ Big DyeTM Terminator v.3.0 Ready Reaction Cycle sequencing Kit (Applied Biosystems, Bleiswijk, The Netherlands) and an ABI PRISM™ 3700 Genetic Analyzer (Applied Biosystems). Contigs were assembled using the forward and reverse sequences with the program SeqMan from the LaserGene package (DNAstar Inc., Madison, USA). The resulting sequences showed homology with the *C. albicans*-type strain CBS 562, not with other *Candida* species-type strains (GenBank Accession numbers, KT271767, KT271768, and KT271769). Multilocus sequence typing was done as previously described by Bougnoux et al. [3] and resulted in a new MLST-type that was given number Diploid Sequence Type number DST2414 (ATT1: 4; ACC1: 7; ADP1:130; MPB1:142; SYA1194; VPS13:51; ZWFb1:254; http://calbicans.mlst.net/). A BLASTn search was performed for the concatenated sequences including all seven loci and a total of 2874 base pairs, with DTS2414 being the query comparing with all 3194 DST-types present in the Candida MLST database. The top-20 results were then aligned with ClustalW using MEGA 6, and a neighbor-joining tree was constructed (Fig. 2) indicating that the five closest MLST-types belong to isolates from various sources and at least two different countries in Europe [4]. The identification of the isolates was also confirmed by MALDI-TOF–MS analysis according to methods described by Cendejas-Bueno et al. [5]. Four single colonies of this isolate yielded reliable and high confidence scores that allowed correct species identification. Using the CBS-KNAW in-house MALDI-TOF–MS libraries, each colony was recognized as *C. albicans* with log score values >2.2.

**Fig. 2:**
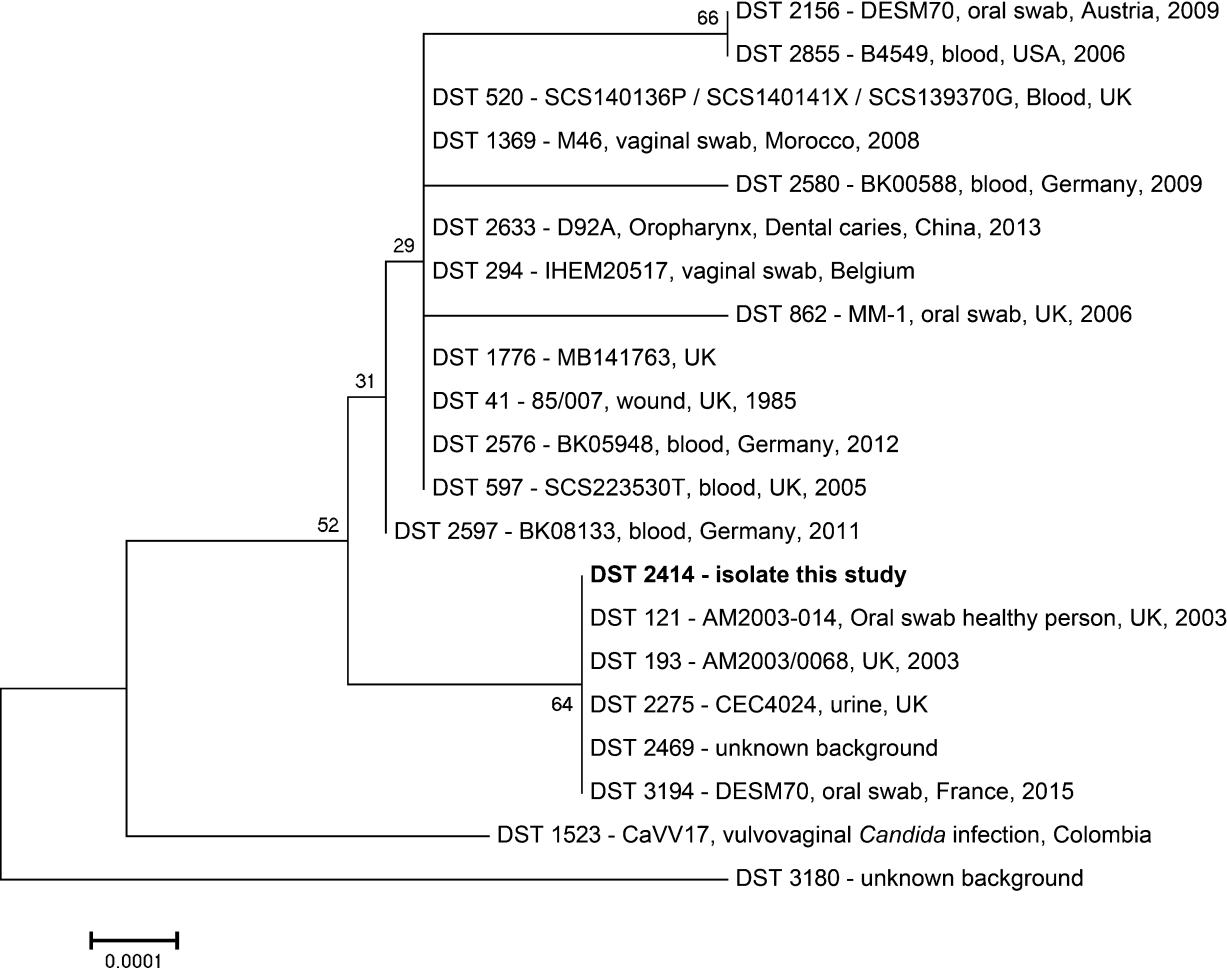
Neighbor-joining tree with 1000 bootstrap replicates of the 20 most similar MLST sequence types (DST-types) with each line indicating DST-type, isolate name, source, country, and year of isolation, if available

The definite diagnosis in this dog was disseminated infection with a *C. albicans* with a novel MLST profile. *Candida* spp., in particular *C. albicans* and *C. parapsilosis*, can be isolated from the ear canals, nose, oral cavity, and anus of clinically healthy dogs and are thus considered commensals of the canine mucosae [6, 7]. Disturbance of the endogenous microflora by antibiotic or immunosuppressive therapy or disruption of the normal cutaneous or mucosal barriers by surgery, indwelling catheters or trauma facilitates entrance of *Candida* spp. to the body [7]. In humans, clinical mucocutaneous *Candida* infections are associated with a high morbidity and invasive candidiasis with a high mortality, with *C. albicans* being the predominant causative species [8, 9]. An impaired cellular immunity and a lack of T cells or neutrophils increase susceptibility to disseminated infection with *Candida* spp. [7, 9–11]. Inherited or acquired disorders of phagocytes have been associated with systemic candidiasis in humans, whereas inherited or acquired disorders of T lymphocytes are associated with chronic mucocutaneous candidiasis [11, 12]. In human patients, cutaneous candidiasis rarely develops into disseminated candidiasis [9]. Similarly, in dogs, progression of a localized infection into a systemic one has not been reported, but reports on (muco)cutaneous [13] and disseminated candidiasis [14–20] in this species are scarce anyway. In the few case reports so far, however, at least one predisposing factor was present or suspected (Table 1), which was not the case in this patient. It should be noted that the dog was successfully treated after being diagnosed with lip and periorbital demodicosis and bilateral ceruminous otitis externa at an age of 8 months, both relatively common in young dogs [21–23].

**Table 1:** Case reports so far on systemic candidiasis in dogs

*Candida* spp.	Determination					Predisposing factor	References
	culture	histo	cyto	IHC	TEM		
*Candida* spp.	–	+	+	+	–	Infection with canine parvovirus	Rodríquez et al. [14]
*Candida* spp.	+	+	+	–	–	Hyperadrenocorticism, diabetes mellitus	Heseltine et al. [15]
*Candida* spp.	–	+	+	+	+	Suspected: antibiotics	Brown et al. [16]
*Candida albicans*	–	+	+	+	–	Suspected: antibiotics	Kuwamura et al. [17]
*Candida* spp.	+	+	+	–	–	Peritonitis secondary to enterotomy site dehiscence	Rogers et al. [18]
*Candida albicans*	+	+	+	+	–	Mesenteric mast cell tumor with metastases	Matsuda et al. [19]
*Candida* spp.	+	–	+	–	–	Peritonitis secondary to enterectomy site dehiscence	Ong et al. [20]

At least one predisposing factor was present or suspected; *histo* histopathology, *cyto* cytopathology, *IHC* immunohistochemistry, *TEM* transmission electron microscopy

Primary susceptibility to yeast infections and demodicosis may be associated with endocrinopathies such as hyperadrenocorticism, diabetes mellitus, and hypothyroidism [23–26]. However, based on medical history, clinical signs, and blood tests, these disorders were considered highly unlikely. In addition, a primary (hereditary or congenital) immunodeficiency disorder, i.e., a defect in the cell-mediated immune system, is not likely because of the age of the dog. A secondary (acquired) immunodeficiency disorder, i.e., suppression of the cell-mediated immune system or a defect in phagocyte function, seems more likely, but the patient had not previously shown indicative signs.

The route of entrance of the *C. albicans* in this dog remains unknown. There were no signs of disruption of the gastrointestinal, respiratory, or urogenital mucosae, but the most likely explanation is that *C. albicans* somehow passed the mucosal barrier and was subsequently distributed hematogenously either directly establishing numerous local infections or indirectly from an initial submucosal local infection. Small blood vessels might have been embolized by yeasts leading to micro-abscesses, as has been described by Rodríguez et al. [14] in skin, lungs, myocardium, liver, kidney, brain eyes, and skeletal muscles of a puppy with a, probably facilitating, parvovirus infection.

In conclusion, the presented canine patient had no apparent predisposing conditions facilitating the development of disseminated candidiasis, suggesting that the agent itself was the primary cause. Formation of germ tubes in serum is the common method for identifying *C. albicans*, but it has been shown that 5% of *C. albicans* strains are known to lack this trait [27, 28]. Albeit its low identification in API 20 C AUX, the molecular identification by ITS, including material from a biopsy, and MALDI-TOF–MS was clear. The novel MLST profile may indicate that this strain possesses unusual properties regarding clinical infestations [29, 30]. Future MLST typing and investigation of virulence attributes can provide further evidence whether this MLST-type is associated with clinical cases of disseminated candidiasis without an apparent predisposing condition.
